# Solutions to Address Inequity in Diabetes Technology Use in Type 1 Diabetes: Results from Multidisciplinary Stakeholder Co-creation Workshops

**DOI:** 10.1089/dia.2021.0496

**Published:** 2022-05-31

**Authors:** Shivani Agarwal, Gladys Crespo-Ramos, Stephanie L. Leung, Molly Finnan, Tina Park, Katie McCurdy, Jeffrey S. Gonzalez, Judith A. Long

**Affiliations:** ^1^ Division of Endocrinology, Diabetes, and Metabolism, Fleischer Institute for Diabetes and Metabolism, Albert Einstein College of Medicine-Montefiore Medical Center, Bronx, New York, USA.; ^2^ NY-Regional Center for Diabetes Translation Research, Albert Einstein College of Medicine, Bronx, New York, USA.; ^3^ Ferkauf Graduate School of Psychology, Yeshiva University, Bronx, New York, USA.; ^4^ Diagram LLC, New York, New York, USA.; ^5^ Division of General Internal Medicine, Perelman School of Medicine, University of Pennsylvania, Philadelphia, Pennsylvania, USA.; ^6^ Corporal Michael J. Crescenz VA Medical Center, Philadelphia, Pennsylvania, USA.

**Keywords:** Inequity, Disparities, Race–ethnicity, Minority, Diabetes technology, Type 1 diabetes

## Abstract

**Background:**

Racial–ethnic inequity in type 1 diabetes technology use is well documented and contributes to disparities in glycemic and long-term outcomes. However, solutions to address technology inequity remain sparse and lack stakeholder input.

**Methods:**

We employed user-centered design principles to conduct workshop sessions with multidisciplinary panels of stakeholders, building off of our prior study highlighting patient-identified barriers and proposed solutions. Stakeholders were convened to review our prior findings and co-create interventions to increase technology use among underserved populations with type 1 diabetes. Stakeholders included type 1 diabetes patients who had recently onboarded to technology; endocrinology and primary care physicians; nurses; diabetes educators; psychologists; and community health workers. Sessions were recorded and analyzed iteratively by multiple coders for common themes.

**Results:**

We convened 7 virtual 2-h workshops for 32 stakeholders from 11 states in the United States. Patients and providers confirmed prior published studies highlighting patient barriers and generated new ideas by co-creating solutions. Common themes of proposed interventions included (1) prioritizing more equitable systems of offering technology, (2) using visual and hands-on approaches to increase accessibility of technology and education, (3) including peer and family support systems more, and (4) assisting with insurance navigation and social needs.

**Discussion:**

Our study furthers the field by providing stakeholder-endorsed intervention ideas that propose feasible changes at the patient, provider, and system levels to reduce inequity in diabetes technology use in type 1 diabetes. Multidisciplinary stakeholder engagement in disparities research offers unique insight that is impactful and acceptable to the target population.

## Introduction

Mounting evidence from our group and others underscores health inequity between Black and Hispanic versus White people with type 1 diabetes, including nearly two percentage point higher mean glycated hemoglobin (HbA1c) values, twice the risk of diabetic ketoacidosis and hospitalization, and 1.5 times higher risk of mortality.^1–3^ Advanced diabetes technologies, such as insulin pumps and continuous glucose monitors (CGMs), facilitate diabetes self-management, improve quality of life, and improve HbA1c up to one percentage point—showing clear potential to modify long-term outcomes and reduce disparities.^4–8^

The proliferation of technological options, increased ease of use, and continued high-quality evidence of glycemic benefit have led professional societies to recommend diabetes technology to become a standard part of care for patients with type 1 diabetes.^9,10^ However, recent reports demonstrate stark racial–ethnic disparities in technology use, with nearly two to four times higher rates of CGM and insulin pump use in White versus Black and Hispanic people with diabetes.^1,11–14^ As evidence of inequity in technology use grows,^11,12,15–19^ there remains a paucity of well-informed interventions to increase use in underserved populations with type 1 diabetes.

Underserved populations have unique health care experiences and social needs that may be limiting diabetes technology use. Challenges from social determinants of health,^1,20–28^ low social support,^29–32^ structural racism,^33–36^ and inequities in health care delivery^1,11,12,15,17,18,37^ can contribute to fractured care, lost opportunities for building rapport with providers, and inability to follow traditional diabetes care guidelines.

Most studies highlighting racial–ethnic inequity in diabetes technology use cite socioeconomic status (SES) and insurance as main drivers, but emerging evidence has not fully substantiated this long-held belief. We found in a national study of 300 young adults with type 1 diabetes that White young adults were two and four times more likely to use diabetes technology than Hispanic and Black young adults, respectively,^21^ despite state insurance coverage for technology at the majority of study sites. We further showed that factors related to demographics, insurance, social determinants of health, health care, and diabetes self-management did little to explain large differences in insulin pump and CGM use between White, Black, and Hispanic young adults with type 1 diabetes.^11^

Our findings were consistent with data from the Type 1 Diabetes Exchange registry of 25,000 people in which Black children and adults were found to use diabetes technology at 50% the rate of White children and adults, regardless of insurance status or income level.^12,13^ Understanding and addressing unmet patient, provider, and system needs are necessary if we want to address disparities in technology use.

In a qualitative study from our group exploring technology disparities with 50 Black and Hispanic young adults with type 1 diabetes, participants noted a lack of shared decision making and opportunities for discussion of technology with providers. Moreover, these young adults stated that when they felt heard, respected, supported, and helped with social needs, they were more likely to use diabetes technology.^15^ In another study of 86 adults with type 1 diabetes at federally qualified health centers, similar provider interactions and system-level issues prevented use of technology.^19^ These findings are consistent with studies that found physicians unconsciously and preferentially prescribed diabetes technology for youth and adult patients with type 1 diabetes who exhibited higher health literacy, SES, and lower HbA1c values.^1,12,38,39^

Prior qualitative studies in underserved patients with type 1 diabetes have highlighted key solutions to addressing inequity in technology use, by improving patient–provider interactions and removing system-level barriers.^15,19^ In an effort to include health care providers in both primary and specialty care in intervention development, and to better understand how to implement proposed solutions to increase acceptability, efficacy, and sustainability among all stakeholders, we employed user-centered design to ask stakeholders to co-create solutions and provide more detail on proposed interventions. We leveraged an approach that is interactive, emphasizes collaboration and co-creation among stakeholders, and leverage multiple viewpoints from people to optimize acceptability and implementation.

## Methods

### Stakeholder participants

Stakeholder characteristics are summarized in Supplementary Table S1. Patient participants were included if they had a clinical diagnosis of type 1 diabetes, were of ages 18–30 years, self-reported non-Hispanic Black or Hispanic race–ethnicity, had ability to participate in collaborative conversation with others, and had recently started on diabetes technology in the past 18 months. The decision was made to engage recent technology users to elicit perspectives of patients while off and on technology, and to ensure that onboarding and new user experiences were recent enough to provide insights relating to decision making about initiation of technology use. A wide variety of potential technologies was included such as CGM only, pump only, and automated insulin delivery (AID).

Patient participants were recruited from the Bronx, NY, which is one of the poorest and most underserved counties in the United States.^44^ Patients were mainly recruited for the prototyping phase given our and other previously published studies on patient-perceived facilitators and barriers.^15,16,18,19,41^

Provider participants provided a broad range of expertise in type 1 diabetes, racial–ethnic disparities, and/or social determinants of health. Given large gaps in the literature describing provider barriers, facilitators, and solutions on increasing diabetes technology use in underserved populations, we oversampled providers with a wide variety of experiences and from across the United States. Providers were pediatric or adult endocrine physicians/nurse practitioners, general pediatricians or internal medicine physicians, pediatric or adult psychologists, diabetes educators, or community health workers.

### Research team

The multidisciplinary research team from Albert Einstein College of Medicine included expertise in diabetology, internal medicine, type 1 diabetes psychology, social determinants of health, and public health. We employed a health care design firm, Diagram LLC, which included expertise in user-centered design methods, health care design, graphic design, and health care corporations.

### User-centered design workshops

Together with Diagram LLC, we developed virtual workshops to convene stakeholders and participate in activities that fostered cross-disciplinary discussion and collaboration of ideas. User-centered design principles were employed, such as empathizing, defining, ideating, and prototyping. The goal of empathizing is to better understand the problem and work through deeper comprehension of its various facets. The goal of defining is to synthesize all of the existing data and define the scope and levels of the problem. For empathizing and defining, our research team and Diagram Inc. reviewed our in-depth qualitative data examining diabetes technology use, facilitators, barriers, and proposed solutions from >50 underserved young adults with type 1 diabetes,^15^ as well as other available literature.

For ideating and prototyping, we created and ran workshops where stakeholders used the existing data and their own knowledge of the problem to devise solutions where anything was possible. Workshops were offered in Spanish and English; however, no participants preferred Spanish language workshops despite stating they were bilingual. Patient participants were divided by race–ethnicity (Hispanic and non-Hispanic Black) to foster social cohesion, per patient preference. Patient workshops were broken into three activities.

In the first two activities, patients were asked to confirm problems and facilitators along the technology use journey highlighted in the literature and were given the opportunity to add new information. These ideas were synthesized in real time by workshop moderators from the research team for the third activity where patients brainstormed solutions individually and as a group. After solutions were shared, patients voted on the top 2 ideas, the logistics of which were then further fleshed out with a series of implementation-specific questions in the prototyping phase: (1) who delivers the intervention, (2) what content is covered and what barriers/anticipated outcomes are expected, (3) where will the intervention take place (home, clinic, community, or some combination), (4) when should the intervention take place (synchronous or asynchronous with medical appointments and time of day), and (5) how should it be delivered (virtual, in-person, and combination of both)?

In provider workshops, the first activity included a review of patient priorities and brainstormed solutions, with solicitation of provider barriers and facilitators in the context of patient needs. The second activity consisted of provider-generated intervention ideas elicited through individual and group brainstorming time, followed by the third activity of exploring intervention logistical details. The first two activities covering empathizing, defining, and ideating were heavily focused on for providers given that most of the insights had not been studied before, especially from the viewpoint of primary care, psychologists, educators, or community health workers.

We did not include patients in the provider sessions because of the known power dynamic and our formative study with patients stating they would not feel comfortable ideating with providers present.

### Procedures

Workshops were developed iteratively over the course of 2 months with a careful vetting process by our multidisciplinary research team, with initial drafting and edits made by Diagram LLC and subsequent changes made exclusively by the research team in response to preliminary testing (prototyping) with potential participants and scientific experts. Workshops were rehearsed internally in mock sessions to provide additional feedback and refine content and delivery until the research team unanimously decided that workshops were finalized.

**FIG. 1.**
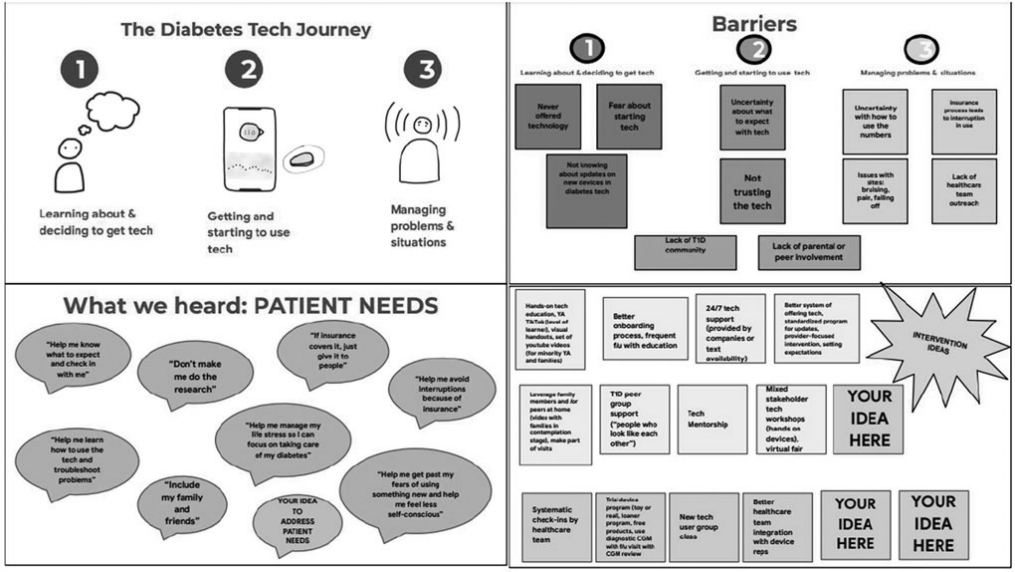
Sample workshop slides demonstrating user-centered design principles: empathizing, defining, ideating, and prototyping/co-creation. T1D, type 1 diabetes; YA, young adults.

Participants were recruited through phone calls and/or email communication. After introduction of the study, informed consent was obtained. Workshops were held on privacy-protected Zoom and recorded for analysis purposes only. This study was approved by the Albert Einstein College of Medicine Institutional Review Board. All participants were provided compensation for their time. A sample compilation of workshop slides is displayed in Figure 1.

### Analysis

Two members of the Diagram team and two members of the Albert Einstein College of Medicine team performed initial thematic analysis and synthesis of results from all workshops. Coding frameworks were devised according to workshop activity and detailed analysis logs kept by the research team. Analysis was done in parallel between the Diagram and Albert Einstein teams, with the entire research team convening several times throughout the analysis process to discuss results and reconcile coding differences. After this iterative coding process, the principal investigator (S.A.) rewatched all recorded workshop sessions and confirmed that results accurately reflected the participant data.

## Results

In all, our team conducted 7 virtual workshops through Zoom including 32 stakeholders: 5 non-Hispanic Black and 7 Hispanic patients with type 1 diabetes, and 20 provider stakeholders from 11 different states. Patients were on a variety of technologies including CGM only (*n* = 6), insulin pump only (*n* = 3), and AID systems (*n* = 3). The workshops were conducted for 7 weeks (from August of 2020 to October 2020).

### Empathizing and defining phase results

#### Patients

Patient stakeholders confirmed much of what has been previously discussed in patient perspective studies on this topic, outlining a linear chronological journey of learning about and getting technology, technology onboarding and starting use, and managing ongoing problems. Barriers included discomfort or fear of new devices as well as needing help with troubleshooting and overall support to sustain technology use, such as education and insurance assistance. Facilitators included family and friend involvement and supportive health care provider teams.

#### Providers

Providers confirmed similar experiences of their patients. In addition, providers detailed their own possible biases, noting real unconscious bias specifically related to the ability of patients to “handle technology.” They detailed several barriers to traditional self-management in their underserved patients that made them hesitant to prescribe technology, such as education gaps, literacy limitations, inconsistent clinic attendance rates, and management of social determinants. Primary care providers stated that other lack of expertise was associated with lower confidence in prescribing technology even when they perceived benefits for patients.

All providers endorsed that their practice and health system structures were not amenable to patient-centered care. Their limited time with patients during appointments prevented them from having the in-depth conversations they knew they needed to fully introduce technology. They further mentioned that they had little to no support with insurance paperwork that prevented them from pursuing technology for many patients. Lastly, they mentioned that the perceived extra outreach they would need to provide to underserved patients to use technology was also a major barrier to prescribing.

Psychologists mentioned the need for peer support and involvement of family. Having safety nets for diabetes support helped with continued use of technology despite hassles. Social workers and community health workers all noted that underserved patients needed extra clinical and administrative support that was not available, especially in adult health care systems. Importantly, most health care providers did not feel equipped to screen for or manage social needs necessary for underserved patients to successfully initiate or continue use of technology.

### Ideating and prototyping phase results

**FIG. 2.**
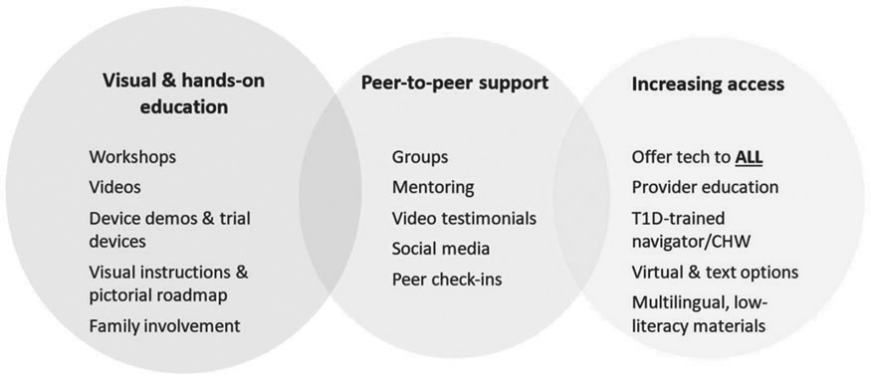
Key elements of a diabetes technology equity intervention discussed by multidisciplinary stakeholders. CHW, Community Health Worker.

Stakeholders co-created and discussed many solutions to increase technology use in underserved populations with type 1 diabetes, displayed in Figure 2. Interventions fell into several categories, including (1) visual and hands-on education (helping people to understand how everything works and what to expect), (2) peer support (offering practical tips and advice along with emotional support from people with similar lived experiences), and (3) increased access to devices (clearing administrative/insurance and social barriers, meeting people where they are).

For (1) education, stakeholders focused on demystifying technology devices by employing hands-on visual aids in conversations, such as dummy devices or other pictorial representations of products, as well as trial devices. For (2) peer support, stakeholders suggested peer-led workshops, “peer technology mentors,” use of social media campaigns, and inclusion of family members in conversations surrounding technology even when not present for medical visits. For (3) increased access to devices, stakeholders suggested instituting more equitable systems of offering technology, such as removing the provider from having to offer information on devices and instead relying on waiting room marketing materials or creating a specialized front office role for a “technology specialist/consultant.” Stakeholders also suggested a checklist in the electronic medical record to keep track of whether providers had offered technology information to all eligible patients.

In addition, provider stakeholders discussed needing a new role in clinic to implement the technology intervention instead of tasking an existing staff member. Arguments in favor of a new role described benefits of preventing burnout in clinical staff, prime focus on technology, and the opportunity to hire a staff member of color who had shared lived experiences with patients. Arguments against a new role included risk to continuity and consistency, and concerns with integration into the health care team.

Community Health Workers (CHWs) offered themselves as the best interventionists, citing several advantages over other care team members, including (1) not being part of the “system” of care, (2) having direct community experience and firsthand understanding of patient culture and values, (3) ability to make home visits if needed, (4) having more time for outreach and social support, and (5) expertise in social needs screening and social service linkage to remove barriers to technology use. Community health workers also mentioned that recent increased comfort with mobile health strategies and documentation in the electronic medical record due to the COVID-19 pandemic gave them confidence that they could conduct the intervention virtually and become diabetes technology experts.

**FIG. 3.**
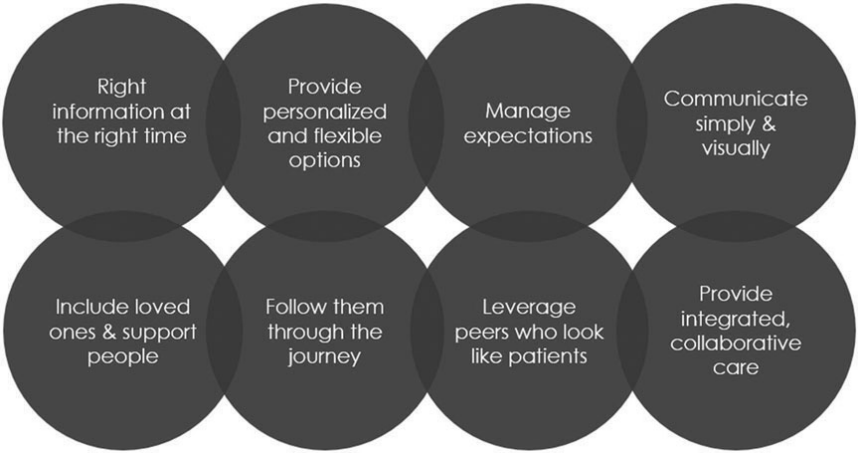
Program principles of interventions to increase diabetes technology use in underserved populations with type 1 diabetes.

Lastly, stakeholders discussed program principles of interventions that would enable success, displayed in Figure 3. These included giving the right information at the right time, providing accessible information including managing expectations, leveraging support networks, creating collaborative technology care, and supporting patients throughout the journey of technology use.

## Discussion

Our study highlights stakeholder-created multilevel solutions to increase use of diabetes technology for underserved populations with type 1 diabetes. Through our innovative approach, we were able to leverage the breadth and depth of new stakeholder views. Top intervention ideas included developing more accessible education using low-literacy visual and interactive materials, instituting equity in offering technology, leveraging peer and family support for initiating technology, and providing more insurance support to practices. User-centered design methodology is a unique and efficient tool in disparities research to elicit stakeholder lived experiences and to develop new collaborative ideas on solutions to address disparities.

Multiple studies in populations with type 1 diabetes have highlighted the role of the health care provider as the “gateway” to diabetes technology.^15,18,19^ Some studies suggest that overt or unconscious bias may be occurring and manifesting itself as lack of shared decision making and microaggressive language during medical encounters.^18,33,42^ Unconscious bias may degrade the patient–provider relationship and trust necessary for patients to accept new treatments, such that patients may be rejecting diabetes technology when offered.^43–45^ In our prior published study with patients, provider behaviors that instilled confidence in new technology use included optimism, tailoring of information, and clinical expertise.

Stakeholders commented that provider bias had to be addressed, but that bias training would not suffice. One study of an intervention to change provider-level biases in diabetes care using cultural competency training and race-stratified performance reports demonstrated that although clinicians acknowledged disparities in their patients' diabetes control, this knowledge alone did not empower them to address the complex root causes of disparities.^53^ Stakeholders discussed that the approach to educate and discuss technology had to be changed dramatically to include more hands-on and visual instructions and demos to bypass bias. Partnering with device companies to obtain demos and device trials has potential to demystify technology for patients and enhance practice ability to make technology feel more accessible. If there is a way to avoid marketing influence, partnerships with industry could provide practices with staff training and onboarding onto devices, and enhance provider ability to offer and support technology use.

In addition, training opportunities are needed to allow providers to role play technology introductory conversations to catch any language that inadvertently promotes implicit bias. Low-literacy visual educational aids on diabetes technology devices are available, some in English and Spanish, but are used sparingly in practice currently.^46–48^ Thus, simple low-touch interventions using readily available resources may be within reach for many practices and could be highly effective.^49^

While health care provider behavior is a key factor in technology use among underserved populations and needs to be a focused part of any intervention,^50–52^ it may not be enough. Stakeholders mentioned that system-level interventions were needed to eliminate current practices that make providers prone to prescribing biases. They discussed that instituting a mission of equity into practices was needed that both standardized care approaches and offered specialized tailoring to underserved populations.

It is also well known that patients struggling with social determinants of health require extra support, but receive lower quality care and suffer from worse health outcomes.^54,55^ Provider stakeholders noted specifically that they lacked the expertise to assess or help manage social needs that act as barriers to technology use. CHW stakeholders noted that they would be optimal in helping to assess social needs and introduce technology in a culturally responsive and less time-pressured way. Thus, the most effective solutions may require practice transformations and inclusion of other team members besides health care providers who are skilled in assessing and managing social needs.

Lastly, stakeholders emphasized the positive influence on technology use of extra support from friends and family. Multiple studies across diabetes and in other fields have demonstrated that inclusion of diabetes support networks in medical care results in higher engagement, improved medical and psychological outcomes, and better cultural competency.^56,57^ Thus, across the lifespan, it may be imperative to include family members and other support systems for diabetes technology initiation and management, especially given the real-time and complex self-management demands a new technological treatment may require.

Practices can develop informal peer-to-peer mentorship programs or connect patients with social media communities that are connected to technology to normalize and contextualize the use of devices for diabetes management, especially if patients can see others “like them.” Nevertheless, such platforms can provide misinformation, and any clinical advice should be directed to the health care provider.

Our study has several limitations. Given the intensive resources used in this methodology, it was not possible to increase our number of participants. To balance potential biases, we chose to leverage prior published qualitative results that represented >100 underserved patients with T1D to prioritize enrollment of a larger number and variety of providers who have largely been missing in the literature thus far. We also amplified views from stakeholders by encouraging collaborative real-time intervention development in our workshops, which provided unique and in-depth insights that have not been offered as of yet. We added rigor to our analytic approach by standardizing our analysis and by performing multiple cycles using multiple coding experts from a health care design company and our research team.

This study used a new approach to elicit unique cross-disciplinary solutions to increase diabetes technology use among underserved populations with type 1 diabetes. With our methodology, we provide researchers and clinicians with a suite of well-informed interventions that have been voted on by stakeholders as highly acceptable and impactful. Regardless of intervention, stakeholders emphasized the importance of changing the system and not only relying on the provider to change practice behaviors in a system that does not facilitate change. With the current wave of diversity, equity, and inclusion initiatives sweeping across academic medical centers in the United States,^52,58^ there is potential to use this study's data to transform the way we care for our underserved type 1 diabetes populations.

## Supplementary Material

Supplemental data
