# Supplementary material for: Solutions to Address Inequity in Diabetes Technology Use in Type 1 Diabetes: Results from Multidisciplinary Stakeholder Co-creation Workshops
Source: Diabetes Technol Ther. 2022 May 31;24(6):381–9. doi: 10.1089/dia.2021.0496 (PMC9208861; doi:10.1089/dia.2021.0496)
Supplement: Supplemental data [file Supp_TableS1.docx]

**Supplemental Table: Stakeholder Characteristics**

| Mean (SD) or n | **Provider** **Stakeholders**  (n=22)  *From: NY, MD, PA, CT, FL, OH, IL, TX, OR, CA* | Mean (SD) or n | **Patient Stakeholders**  (n=10)  *(5 LatinX, 5 NH Black)* |
| --- | --- | --- | --- |
| Age (years) | 46.4 (12.3) | Age (years) | 22.2 (5.6) |
| Gender (female) | 14 | Gender (female) | 9 |
| Years of experience | 12.1 (10.5) | Diabetes Duration (years) | 10.7 (7.2) |
| Endocrinology (MD or NP) | 9 | HbA1c during study (%) | 8.0 |
| Primary Care (MD or NP) | 4 | Treatment Regimen |  |
| T1D Psychologist | 5 | CGM + MDI | 4 |
| Community Health Worker | 4 | Insulin pump only | 1 |
| Adult | 8 | CGM + Insulin pump (not automated) | 3 |
| Pediatric | 10 | Automated Insulin Delivery | 2 |
